# Complete Genome Sequence of Human Echovirus 20 Strain 812/YN/CHN/2010, Associated with Severe Hand-Foot-and-Mouth Disease

**DOI:** 10.1128/genomeA.00486-17

**Published:** 2017-06-08

**Authors:** Yilin Zhao, Haihao Zhang, Hongbo Liu, Hao Sun, Xiaoqin Huang, Zhaoqing Yang, Shaohui Ma

**Affiliations:** a Institute of Medical Biology, Chinese Academy of Medical Sciences, and Peking Union Medical College, Kunming, People’s Republic of China; b Yunnan Key Laboratory of Vaccine Research Development on Severe Infectious Disease, Kunming, People’s Republic of China

## Abstract

Human echovirus 20 (E-20) belongs to the *Human enterovirus B* (HEV-B) species and is often detected in nonpolio enterovirus cases of acute flaccid paralysis. We determined the complete genome of strain 812/YN/CHN/2010, isolated from a child with severe hand-foot-and-mouth disease in Yunnan, China, in 2010.

## GENOME ANNOUNCEMENT

Human echovirus 20 (E-20) belongs to the *Human enterovirus B* (HEV-B) species of the family* Picornaviridae* (1). Echoviruses include 28 serotypes and the genome consists of a 5′ untranslated region (UTR), structural polypeptide P1, nonstructural polypeptides P2 and P3, and a 3′ UTR. Echoviruses can cause a variety of human diseases, such as asymptomatic, acute flaccid paralysis, hand-foot-and-mouth disease (HFMD), and aseptic meningitis and encephalitis (2, 3). Although E-20 is mostly associated with asymptomatic or mild infections, it is occasionally detected in nonpolio enterovirus cases of acute flaccid paralysis (4). E-20 is one of the most commonly isolated echovirus serotypes, yet to date, only two whole genomes of E-20 are available in GenBank, one from the prototype JV-1 strain (AY302546) isolated from the United States in 1956 and the other from the KM-EV20-2010 (KF812551) strain isolated from a patient with hepatitis in 2010 (5).

The E-20 812/YN/CHN/2010 strain was isolated in 2010 from a 10-month-old child during HFMD surveillance in the Yunnan Province. The patient was diagnosed with severe HFMD and a stool specimen collected from the patient was detected positive for E-20 by reverse transcription-PCR (RT-PCR) and BLAST (http://www.ncbi.nlm.nih.gov/BLAST/). Human embryonic lung diploid fibroblast (KMB17) cell lines were used to isolate the virus. We first determined the complete genomes of the E-20 strains isolated from one patient suffering from HFMD in Yunnan, China.

After viral RNA was extracted from cell culture supernatants, the partial VP1 gene was amplified by the one-step RT-PCR method with primer pairs 222 and 224, and BLAST was further used to confirm the serotype of the isolate. Primers for amplifying and sequencing the whole genome were designed as described previously (5, 6). Pairwise alignment of the sequences was performed using Geneious Basic 5.6.5 software. The full-length genome of the 812/YN/CHN/2010 strain consisted of 7,398 nucleotides (nt), composed of a 5′ UTR of 744 nt followed by an open reading frame that encodes a polyprotein of 2,183 amino acids and a 3′ UTR of 102 nt. The contents of A, U, G, and C were 28.02%, 23.94%, 24.76%, and 23.28%, respectively, with G+C contents of 48.04%. The strain shared 80.0% and 99.5% nucleotide similarity with prototype strain JV-1 and KM-EV20-2010 strain in the complete genome sequence, respectively, while, the amino acid sequences were highly conserved (96.4% to 97.3%).

### Accession number(s).

The whole-genome sequence of the 812/YN/CHN/2010 strain described in this study was deposited in the GenBank database under accession number KX060810.
